# The Lung Microbiome: New Principles for Respiratory Bacteriology in Health and Disease

**DOI:** 10.1371/journal.ppat.1004923

**Published:** 2015-07-09

**Authors:** Robert P. Dickson, Gary B. Huffnagle

**Affiliations:** Division of Pulmonary and Critical Care Medicine, Department of Internal Medicine, University of Michigan Medical School, Ann Arbor, Michigan, United States of America; The University of North Carolina at Chapel Hill, UNITED STATES

## Introduction

The principles of respiratory microbiology are being re-evaluated and re-written, starting with the debunked myth of lung sterility. The “terrain” of the respiratory ecosystem differs—anatomically and physiologically—from that of other mucosal sites, and changes dramatically in illness, when the dynamic homeostasis between host and microbiome is disrupted. Researchers are only just beginning to understand the contribution of viruses, phages, and fungi to the lung microbiome; thus, we have restricted our discussion to the bacterial microbiota of the lungs.

## The Lungs Are Not Sterile

The notion that the lungs are sterile is still frequently stated in textbooks, virtually always without citation. This claim, if true, would be extraordinary. Bacteria are remarkably diverse and adaptable; as such, there is virtually no environmental niche on earth so extreme (in oxygen, pH, hydrophobicity, temperature, salinity, predators, nutrient scarcity, etc.) that bacterial communities cannot been found [1]. It would be remarkable if one of the rare bacteria-free environments on this planet was the warm, moist mucosa found inches below the oral cavity, a bacteria-rich environment over which there is a constant flow of bacteria-laden air, microaerosols, and fluids. Numerous studies, dating back nearly a century, have demonstrated that microaspiration is common in healthy, asymptomatic subjects [2–5], and knowledge of the bacterial content of inhaled air is as old as germ theory itself [6]. Since the first culture-independent report of the healthy lung microbiome [7], more than 30 published studies using molecular techniques for bacterial identification have found evidence of bacteria in the lower airways. No modern study has found evidence of their absence.

## Mucosal Biology: The Lungs Are Not the Gut

While the gut and lungs are both mucosa-lined luminal organs with a shared embryological origin, their gross and micro-anatomical features are quite distinct, yielding marked differences in the composition and population dynamics of their microbiota. In the absence of vomiting or esophageal reflux, migration of microbes in the digestive tract is unidirectional (from the mouth to the anus), and is serially interrupted by widely varying physical and chemical barriers. In order for an orally introduced microbe to immigrate into the cecum, it must endure the acidic pH of the stomach (~2.0) and the alkaline pH of the duodenum (~8.0) and compete for resources with a densely populated resident microbiome. By contrast, movement of air, mucus, and microbes in the lung is bidirectional, with no physical barrier between the larynx and the most distal alveolus. Thus the microbiome of the lungs is more dynamic and transient than that of the lower gastrointestinal tract. While the gastrointestinal tract is of uniform temperature (37°C) throughout its entire 9 meters of length, the mucosa of the respiratory tract (a short half-meter in length) represents a gradient from ambient temperature at the point of inhalation to core body temperature in the alveoli [8]. Unlike the gut, the lung environment is oxygen-rich. Though the trachea and bronchi are, like the gut, lined with the heavily glycosylated proteins of secreted mucus, the vast majority of the lung’s surface area is lined with lipid-rich surfactant, which has bacteriostatic effects against select bacterial species [9]. Bacterial density in the airways is quite modest, comparable to that of the duodenum [7], orders of magnitude less than that of the large intestine; thus inter-bacterial metabolic interactions are markedly different. Finally, the gut and lungs differ in the character of host–bacterial interactions. Luminal IgA levels are far higher in the gut, while the lungs exhibit far more extraluminal interactions between bacteria and host leukocytes (alveolar macrophages). Together, these markedly divergent environmental conditions result in correspondingly divergent microbial communities.

## The Lung Microbiome Is Determined by Three Ecological Factors

**Fig 1:**
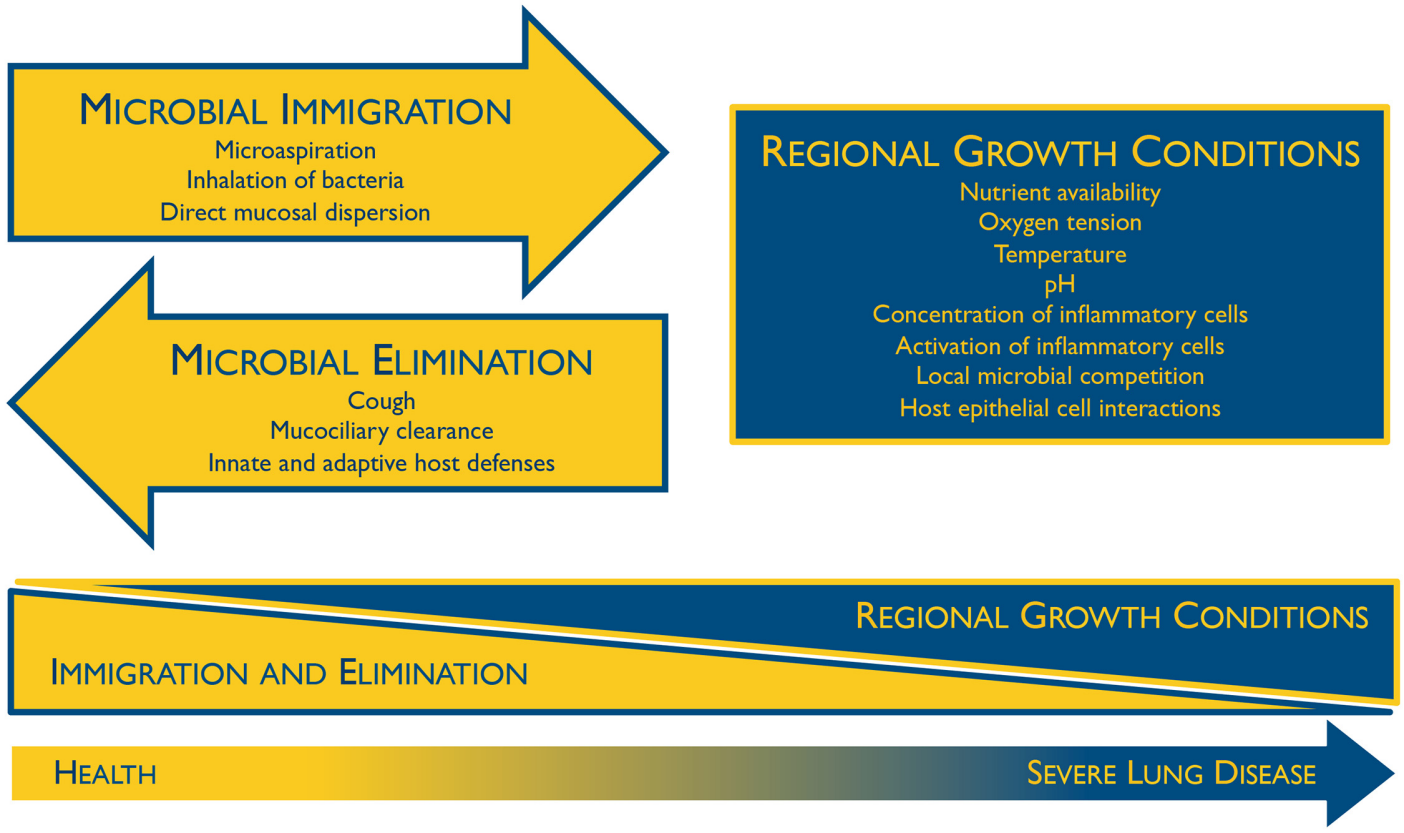
Ecological determinants of the respiratory microbiome. The constitution of the respiratory microbiome is determined by three factors: microbial immigration, microbial elimination, and the relative reproduction rates of its members. In health, community membership is primarily determined by immigration and elimination; in advanced lung disease, membership is primarily determined by regional growth conditions. Adapted from Dickson 2014 [10].

The composition of the lung microbiome is, by first principles, determined by the balance of three factors (Fig 1) [10]: (1) microbial immigration into the airways, (2) elimination of microbes from the airways, and (3) the relative reproduction rates of its community members, as determined by regional growth conditions. Any change in the microbiome—within an individual or across disease states—must be due to some perturbation in these factors. Sources of microbial immigration include the inhalation of air (which contains 10^4^–10^6^ bacteria/mm^3^ even before reaching the bacteria-dense upper airways [11]), subclinical microaspiration of upper respiratory tract contents [2–5], and direct dispersal along airway mucosa. Microbial elimination is driven by mucociliary clearance, cough (which is frequent even among healthy subjects [12]), and host immune defenses (both innate and adaptive). The environmental conditions that determine regional growth conditions in the lungs include both those common to all environmental niches (e.g., nutrient availability, temperature, pH, oxygen tension) as well as the abundance and activation state of host inflammatory cells. In health, these conditions are generally inhospitable for bacterial growth, resulting in relatively little bacterial reproduction; thus the primary determinant of the lung microbiome in health is the balance of immigration and elimination [13–15]. However, during disease, the regional growth conditions of the lungs change dramatically, creating permissive niches for selective bacterial reproduction. The long-recognized phenomenon of bacterial colonization in advanced lung disease reflects the enriched growth of species that are well-adapted to the specific environmental conditions of the injured respiratory tract. The selective reproductive advantage of the lung environment on these community members has overwhelmed the dynamic influence of immigration and elimination on the respiratory ecosystem.

## The Oral Microbiome Is the Primary Source of the Bacterial Microbiota in the Lungs During Health

The ubiquity of subclinical microaspiration of pharyngeal secretions among healthy subjects is a long-established and validated observation [2–5]. Numerous culture-independent studies have since confirmed that the microbiome of the lungs more closely resembles that of the oropharynx than it does competing source communities: inhaled air, the nasopharynx, or the lower gastrointestinal tract via hematogenous spread [15–18]. Both a direct study within individuals and a large population-based model have demonstrated that the nasal microbiome contributes little to lung communities in health [15,16]; the microbiome of the nose more closely resembles that of the skin than that of the lungs. Importantly, this similarity between lung and oral microbiota is evident even when the lung is sampled via a nasally introduced bronchoscope, demonstrating the minimal influence of upper respiratory tract contamination on bronchoscopically acquired specimens [10,19]. The oropharynx produces two liters of saliva per day, a far greater volume of secretions than is produced by the nasal mucosa in health. It is possible (but not proven) that lung and nasal microbial communities converge in times of increased rhinorrhea (e.g., acute viral infections or allergic rhinitis, both of which can provoke exacerbations of lung disease associated with nasal microbes such as *Staphylococcus aureus* and *Moraxella catarrhalis*).

## The Lung Microbiome Changes during Disease

The ecological determinants of the lung microbiome—immigration, elimination, and regional growth conditions—all change dramatically during acute and chronic lung disease [10]. Consequently, the community membership of the lung microbiome is altered in disease states. Of the dozens of studies that have compared the microbiota of diseased lungs with those of healthy subjects, virtually all have found significant differences in community composition [20]. Many have described an increased community richness (number of species) in chronically diseased airways, often with a shift in community composition away from the Bacteroidetes phylum, which dominates the healthy lung microbiome, towards Proteobacteria, the phylum that contains many familiar lung-associated gram-negative bacilli. Baseline differences in lung microbiota have been associated with important clinical features of chronic lung disease: subsequent exacerbation frequency in bronchiectasis [21], mortality in idiopathic pulmonary fibrosis [22], and responsiveness to corticosteroids and antibiotics in asthma [23,24]. Active topics of investigation in the field include (1) whether an altered lung microbiome contributes to disease pathogenesis or is merely a marker of injury and inflammation, (2) whether the lung microbiome can be manipulated therapeutically to change exacerbation frequency or disease progression, and (3) how the diverse and dynamic homeostasis of the lung ecosystem collapses and is dominated by a single dominant pathogen in pneumonia [14,25].

## References

[1] Horikoshi K, Grant WD (1998) Extremophiles: Microbial Life In Extreme Environments: Wiley-Liss.

[2] Gleeson K, Eggli DF, Maxwell SL Quantitative aspiration during sleep in normal subjects. Chest. 1997; 111: 1266–1272. 9149581 10.1378/chest.111.5.1266

[3] Huxley EJ, Viroslav J, Gray WR, Pierce AK Pharyngeal aspiration in normal adults and patients with depressed consciousness. Am J Med. 1978; 64: 564–568. 645722 10.1016/0002-9343(78)90574-0

[4] Quinn LH, Meyer OO The relationship of sinusitis and bronchiectasis. Archives of Otolaryngology—Head & Neck Surgery. 1929; 10: 152.

[5] Amberson JB A clinical consideration of abscesses and cavities of the lung. Bull Johns Hopkins Hosp. 1954; 94: 227–237. 13160680

[6] Pasteur L Expériences relatives aux générations dites spontanées. Comptes Rendus Hebdomadaires des Séances de l'Académie des Sciences D: Sciences Naturelles. 1860; L: 303–307.

[7] Hilty M, Burke C, Pedro H, Cardenas P, Bush A, Bossley C, et al Disordered microbial communities in asthmatic airways. PLoS One. 2010; 5: e8578 10.1371/journal.pone.0008578 20052417 PMC2798952

[8] Ingenito EP, Solway J, McFadden ER Jr., Pichurko B, Bowman HF, Michaels D, et al Indirect assessment of mucosal surface temperatures in the airways: theory and tests. J Appl Physiol. 1987; 63: 2075–2083. 3693240 10.1152/jappl.1987.63.5.2075

[9] Wu H, Kuzmenko A, Wan S, Schaffer L, Weiss A, Fisher JH, et al Surfactant proteins A and D inhibit the growth of Gram-negative bacteria by increasing membrane permeability. J Clin Invest. 2003; 111: 1589–1602. 12750409 10.1172/JCI16889 PMC155045

[10] Dickson RP, Martinez FJ, Huffnagle GB The role of the microbiome in exacerbations of chronic lung diseases. Lancet. 2014; 384: 691–702. 10.1016/S0140-6736(14)61136-3 25152271 PMC4166502

[11] Lighthart B Mini-review of the concentration variations found in the alfresco atmospheric bacterial populations. Aerobiologia. 2000; 16: 7–16.

[12] Munyard P, Bush A How much coughing is normal? Arch Dis Child. 1996; 74: 531–534. 8758131 10.1136/adc.74.6.531 PMC1511564

[13] Dickson RP, Erb-Downward JR, Freeman CM, McCloskey L, Beck JM, Huffnagle GB, et al. Spatial variation in the healthy human lung microbiome and the adapted island model of lung biogeography. Ann Am Thorac Soc. 2015. 10.1513/AnnalsATS.201501-029OC PMC4590020 25803243

[14] Dickson RP, Erb-Downward JR, Huffnagle GB Towards an ecology of the lung: new conceptual models of pulmonary microbiology and pneumonia pathogenesis. Lancet Respir Med. 2014; 2: 238–246. 10.1016/S2213-2600(14)70028-1 24621685 PMC4004084

[15] Venkataraman A, Bassis CM, Beck JM, Young VB, Curtis JL, Huffnagle GB, et al Application of a neutral community model to assess structuring of the human lung microbiome. MBio. 2015; 6. 10.1128/mBio.02284-14 PMC4324308 25604788

[16] Bassis CM, Erb-Downward JR, Dickson RP, Freeman CM, Schmidt TM, Young VB, et al Analysis of the upper respiratory tract microbiotas as the source of the lung and gastric microbiotas in healthy individuals. MBio. 2015; 6. 10.1128/mBio.00037-15 PMC4358017 25736890

[17] Morris A, Beck JM, Schloss PD, Campbell TB, Crothers K, Curtis JL, et al Comparison of the respiratory microbiome in healthy nonsmokers and smokers. Am J Respir Crit Care Med. 2013; 187: 1067–1075. 10.1164/rccm.201210-1913OC 23491408 PMC3734620

[18] Segal LN, Alekseyenko AV, Clemente JC, Kulkarni R, Wu B, Chen H, et al Enrichment of lung microbiome with supraglottic taxa is associated with increased pulmonary inflammation. Microbiome. 2013; 1: 19 10.1186/2049-2618-1-19 24450871 PMC3971609

[19] Dickson RP, Erb-Downward JR, Freeman CM, Walker N, Scales BS, Beck JM, et al Changes in the lung microbiome following lung transplantation include the emergence of two distinct pseudomonas species with distinct clinical associations. PLoS ONE. 2014; 9: e97214 10.1371/journal.pone.0097214 24831685 PMC4022512

[20] Dickson RP, Erb-Downward JR, Huffnagle GB The role of the bacterial microbiome in lung disease. Expert Rev Respir Med. 2013; 7: 245–257. 10.1586/ers.13.24 23734647 PMC4007100

[21] Rogers GB, Zain NM, Bruce KD, Burr LD, Chen AC, Rivett DW, et al A novel microbiota stratification system predicts future exacerbations in bronchiectasis. Ann Am Thorac Soc. 2014;11:496–503. 10.1513/AnnalsATS.201310-335OC 24592925

[22] Molyneaux PL, Cox MJ, Willis-Owen SA, Mallia P, Russell KE, Russell AM, et al The role of bacteria in the pathogenesis and progression of idiopathic pulmonary fibrosis. Am J Respir Crit Care Med. 2014; 190: 906–913. 10.1164/rccm.201403-0541OC 25184687 PMC4299577

[23] Huang YJ, Nelson CE, Brodie EL, Desantis TZ, Baek MS, Liu J, et al Airway microbiota and bronchial hyperresponsiveness in patients with suboptimally controlled asthma. J Allergy Clin Immunol. 2011; 127: 372–381 e371–373. 10.1016/j.jaci.2010.10.048 21194740 PMC3037020

[24] Goleva E, Jackson LP, Harris JK, Robertson CE, Sutherland ER, Hall CF, et al The effects of airway microbiome on corticosteroid responsiveness in asthma. Am J Respir Crit Care Med. 2013; 188: 1193–1201. 10.1164/rccm.201304-0775OC 24024497 PMC3863730

[25] Dickson RP, Erb-Downward JR, Prescott HC, Martinez FJ, Curtis JL, Lama VN, et al Analysis of culture-dependent versus culture-independent techniques for identification of bacteria in clinically obtained bronchoalveolar lavage fluid. J Clin Microbiol. 2014; 52: 3605–3613. 10.1128/JCM.01028-14 25078910 PMC4187760

